# Vaccine-Mediated Mechanisms Controlling Replication of *Francisella tularensis* in Human Peripheral Blood Mononuclear Cells Using a Co-culture System

**DOI:** 10.3389/fcimb.2018.00027

**Published:** 2018-02-07

**Authors:** Kjell Eneslätt, Igor Golovliov, Patrik Rydén, Anders Sjöstedt

**Affiliations:** ^1^Department of Clinical Microbiology, Clinical Bacteriology, and Laboratory for Molecular Infection Medicine Sweden, Umeå University, Umeå, Sweden; ^2^Department of Mathematics and Mathematical Statistics, Umeå University, Umeå, Sweden

**Keywords:** *F. tularensis*, *in vitro* model, human immune response, IFN-γ, TNF, MIP-1β, correlates of immunity

## Abstract

Cell-mediated immunity (CMI) is normally required for efficient protection against intracellular infections, however, identification of correlates is challenging and they are generally lacking. *Francisella tularensis* is a highly virulent, facultative intracellular bacterium and CMI is critically required for protection against the pathogen, but how this is effectuated in humans is poorly understood. To understand the protective mechanisms, we established an *in vitro* co-culture assay to identify how control of infection of *F. tularensis* is accomplished by human cells and hypothesized that the model will mimic *in vivo* immune mechanisms. Non-adherent peripheral blood mononuclear cells (PBMCs) were expanded with antigen and added to cultures with adherent PBMC infected with the human vaccine strain, LVS, or the highly virulent SCHU S4 strain. Intracellular numbers of *F. tularensis* was followed for 72 h and secreted and intracellular cytokines were analyzed. Addition of PBMC expanded from naïve individuals, i.e., those with no record of immunization to *F. tularensis*, generally resulted in little or no control of intracellular bacterial growth, whereas addition of PBMC from a majority of *F. tularensis*-immune individuals executed static and sometimes cidal effects on intracellular bacteria. Regardless of infecting strain, statistical differences between the two groups were significant, *P* < 0.05. Secretion of 11 cytokines was analyzed after 72 h of infection and significant differences with regard to secretion of IFN-γ, TNF, and MIP-1β was observed between immune and naïve individuals for LVS-infected cultures. Also, in LVS-infected cultures, CD4 T cells from vaccinees, but not CD8 T cells, showed significantly higher expression of IFN-γ, MIP-1β, TNF, and CD107a than cells from naïve individuals. The co-culture system appears to identify correlates of immunity that are relevant for the understanding of mechanisms of the protective host immunity to *F. tularensis*.

## Introduction

Tularemia is a severe disease affecting many mammalian species and the etiological agent is the highly virulent bacterium, *Francisella tularensis* (Sjöstedt, 2007). Tularemia in humans is essentially always caused by either of two subspecies, *tularensis* (type A) and *holarctica* (type B), both of which are highly contagious. The former is distinctly more virulent with the potential to cause lethal disease, but there are also numerous descriptions of serious disease caused by type B strains. Tularemia is essentially confined to and reported from many countries of the Northern hemisphere. It is endemic in certain parts of Scandinavia and Turkey, but infrequently reported in most other countries of the world. A human vaccine strain exists, the live vaccine strain (LVS). Vaccination with LVS appears to have made an important contribution for prevention of laboratory-acquired infection, since the number of tularemia cases decreased very significantly among laboratory staff (Burke, 1977). However, despite the efficacious protection observed in the former group, only limited protection was observed when volunteers were subjected to aerosol infection with *F. tularensis* (reviewed by Conlan, 2011). In addition, a lack of understanding of the protective mechanisms has hampered its licensure. Therefore, more efficacious *Francisella* vaccines are needed and an essential basis for such work will be a thorough understanding of immune mechanisms conferring protection against tularemia.

A number of studies have characterized the human memory immune responses resulting from tularemia or tularemia vaccination (Tärnvik et al., 1985; Tärnvik, 1989; Karttunen et al., 1991; Surcel et al., 1991; Sjöstedt et al., 1992; Ericsson et al., 1994; Eneslätt et al., 2011, 2012). In accordance with the intracellular nature of the pathogen, most evidence indicates that cell-mediated immunity (CMI) is the predominant factor contributing to the protective efficacy of the tularemia vaccine (Tärnvik, 1989). The CMI is long-lasting and preserved for at least 25 years after vaccination or natural infection (Ericsson et al., 1994; Eneslätt et al., 2011, 2012). In fact, tularemia offers a unique model for studying the longevity of CMI in humans because it is such a rare disease; in most cases, therefore, re-exposure is very unlikely to be responsible for the persistence of immunity (Eneslätt et al., 2011).

CMI is critically required for efficacious protection against tularemia and, therefore, there is a need to obtain a detailed understanding of how this is effectuated in order to rationally develop future vaccines. Much evidence indicates that protection is carried out via a complex interaction of multiple T cell subsets and other immune mechanisms, rather than a unique immune mechanism (Elkins et al., 2007; De Pascalis et al., 2008, 2012, 2014; Shen et al., 2010; Cowley and Elkins, 2011; Eneslätt et al., 2011, 2012; Ryden et al., 2012). Therefore, simple proliferation assays will not be sufficient to fully delineate the effector mechanisms, but rather assays that closely mimic the *in vivo* situation will be required. Thus, more sophisticated models will be needed to elucidate the protective mechanisms and to identify putative correlates of protection, all of which will be necessary in order to assess vaccine candidates. In this regard, substantial work with the aim to implement and validate *ex vivo* murine infection model systems has been performed to identify effector mechanisms of protective immune responses against *F. tularensis* (Cowley and Elkins, 2003; Cowley et al., 2005; Collazo et al., 2009; Elkins et al., 2011; De Pascalis et al., 2012, 2014; Mahawar et al., 2013; Griffin et al., 2015). Such assays, which measure immune-mediated inhibition of bacterial proliferation and their correlation to specific immunological parameters, allow direct assessments of protective immunity. The relevance of the identified correlates using these assays has to some extent been validated by demonstrating their important roles *in vivo* (Kurtz et al., 2013; Melillo et al., 2013, 2014). A limitation of most of the published work has been the use of the attenuated LVS strain and only few studies using fully virulent *F. tularensis* in the models have been performed (Mahawar et al., 2013; Griffin et al., 2015; Golovliov et al., 2017). An additional caveat is the lack of understanding of how relevant these putative protective correlates are for protection against tularemia in humans. Thus, and in conjunction with the aforementioned need to obtain a thorough understanding of immune mechanisms conferring protection against tularemia, there is a need to develop *in vitro* assays that can be used for the purpose of identifying human immune effector mechanisms that control *F. tularensis* replication and to determine if the mechanisms identified in animal models can be validated.

Previous studies have concluded that the *F. tularensis*-specific T cells are characterized by production of IFN-γ by both CD4 and CD8 T cells that express CCR7 or CD62L (Surcel et al., 1991; Eneslätt et al., 2011). In addition to IFN-γ, intracellular cytokine detection has demonstrated that the responding T cells also are characterized by expression of MIP-1β and CD107a (Eneslätt et al., 2012). In the mouse model of tularemia, numerous studies have demonstrated the important roles of IFN-γ and TNF for the primary as well as the secondary protective immune responses (Anthony et al., 1989; Fortier et al., 1992; Leiby et al., 1992; Conlan et al., 1994; Elkins et al., 1996; Sjöstedt et al., 1996; Cowley et al., 2010). Work has been aimed to identify correlates of immunity by describing cytokine profiles that uniquely identify the proliferative responses of *F. tularensis*-immune individuals (Eneslätt et al., 2011, 2012). In one study, levels of MIP-1β, IFN-γ, IL-10, and IL-5 discriminated vaccinees vs. naïve individuals (Eneslätt et al., 2012). Moreover, secretion of IL-17 has been identified as a characteristic cytokine of the *F. tularensis* memory response. However, if these cytokines are merely correlates of immunity, or also required for the control of infection is unknown.

Validation of tularemia vaccine candidates is challenging since the disease is rarely and irregularly occurring in most countries and therefore, the degree of protection achieved by vaccination will not be possible to evaluate as for most other vaccines (Sjöstedt, 2007). There are examples when human challenge studies have been performed to evaluate vaccine efficacy, e.g., malaria, influenza, and typhoid (Sauerwein et al., 2011; Shirley and McArthur, 2011), however, in view of the high virulence of respiratory infection with *F. tularensis*, it is highly unlikely that such studies will be ethically approved. In addition, correlates of protection conferred by CMI are difficult to identify and absent for most intracellular infections. Collectively, all evidence indicates that the efficacy of a new tularemia vaccine, similar to vaccines protecting against other rarely occurring, serious infections, needs to be assessed as stipulated by the FDA Animal Rule (Snoy, 2010). It states that efficacy can be evaluated by use of animal models only, given that the protective mechanisms of the vaccine are well-understood and thereby can be extrapolated to the human situation. Thus, the implementation of the rule for tularemia vaccines will require that relevant animal models and correlates have been identified in these models, but also that models are established to characterize human correlates of immunity and protection. The latter will have to rely on bactericidal effects as surrogate measures of vaccine efficacy. To initiate the work needed to accomplish this, we here demonstrate that a novel assay based on infection of human adherent peripheral blood mononuclear cells (PBMCs) with either the LVS strain, or the highly virulent SCHU S4 strain, shows that infection can be controlled by the addition of non-adherent PBMC. In addition, the control of *F. tularensis* infection correlated with the expression of IFN-γ, MIP-1β, TNF, and CD107a by CD4 T cells in LVS-infected cultures and with the secretion of IFN-γ and MIP-1β in both LVS and SCHU S4-infected cultures.

## Materials and methods

### Bacterial strains

*Francisella tularensis* LVS was originally obtained from the American Type Culture Collection (ATCC 29684). *F. tularensis* strain SCHU S4 (*F. tularensis* subsp. *tularensis*) was obtained from the *Francisella* Strain Collection of the Swedish Defense Research Agency, Umeå, Sweden. All bacteriological work related to the SCHU S4 strain was carried out in a biosafety level 3 facility certified by the Swedish Work Environment Authority. Before infection, bacteria were grown on modified GC-agar base at 37°C overnight. Formalin-killed bacteria were prepared by incubating LVS or SCHU S4 in 4% paraformaldehyde for 45 min at 37°C followed by three washes in PBS.

### Blood donors

Individuals included in the study had either (i) previously been vaccinated with LVS, henceforth designated vaccinees, or (ii) had no anamnestic data of LVS vaccination, tularemia, or occupational exposure to *F. tularensis*, henceforth designated naïve individuals. All vaccinees had been administered the same lot of LVS, designated NDBR 101, lot no. 11. The mean age and sex distribution of each group was for naïve individuals 38.2 ± 12.9 years (3 females, 8 males) and for vaccinees 49.9 ± 11.2 years (7 females, 4 males). Ethical approvals, 09-181M and 2016/335-31, were obtained from the Regional Ethical Review Board in Umeå, Sweden, and a written informed consent was obtained from all individuals included in the study.

### PBMC collection

Venous blood from donors was collected using CPT-tubes (Becton Dickinson, NJ, USA) and PBMC were prepared according to the manufacturer's recommendations. After washing with 10% of heat-inactivated fetal calf serum in RPMI 1640 (Invitrogen), cells were diluted in culture medium with 10% of heat-inactivated human serum in RPMI 1640. Cells were allowed to recover overnight; cell viability and the cell recovery rate were determined prior to subsequent functional assays.

### Recall stimulation and lymphocyte proliferation assay (LPA)

PBMC were seeded at 2 × 10^5^ cells/well in 200 μL culture medium with 40 μg/mL gentamicin per well in 96-well plates. Cells were stimulated with formalin-fixed LVS and SCHU S4 mixed in equal amounts (ffFt) at final concentrations of 0.1, 0.5 colony forming units (CFU)/PBMC, or without antigen and incubated for 5 days at 37°C in a humidified atmosphere with 5% CO_2_. LPA was assessed by thymidine incorporation in triplicates as previously described (Ericsson et al., 1994).

### Culture system to assess intracellular bacterial replication

PBMC obtained from naïve individuals or vaccinees were separated into adherent and non-adherent cell populations. The adherent population were incubated for 6 days in a 96-well plate at a density of 0.25 × 10^6^ cells/ml and the non-adherent cells were stimulated with 0.5 ffFt/PBMC and incubated for 6 days at a density of 1 × 10^6^ cells/ml. The non-adherent comprise a majority of morphologically similar cells, presumably lymphocytes. However, some of the cells showed an aberrant morphology and presumably were monocytes. Therefore, the non-adherent population also contained some antigen-presenting cells. After washing, adherent cells were infected with LVS or SCHU S4 at an MOI of 10:1 (bacterium-to-adherent cell) for 2 h, washed and incubated for 45 min with culture media containing 40 μg/mL gentamicin. Following two washing steps, non-adherent cells were added at an effector/target ratio of 20:1, and cultures incubated for 72 h. Bacterial counts were determined by lysis of cultures and the number of CFU determined by plating. These MOIs and ratios were found to be optimal in preliminary experiments.

### Multiplex cytokine analysis

Cell culture supernatants, 30 μL/well, were collected from the same cell cultures as used for assessment of intracellular bacterial replication after 72 h of incubation and stored frozen at−80°C. The time point was chosen since levels of several cytokines increased in the supernatants between 24 and 72 h. The supernatants were analyzed using two custom-made multiplex kits and a Bio-Plex 200 system (BioRad Laboratories Inc., Hercules, CA, USA) according to the manufacturer's instructions. A 5-plex kit and 10-fold diluted supernatants were used to determine the levels of MIP-1β, MCP-1, IL-6, IFN-γ, and TNF (high level cytokines), and a 6-plex kit in combination with two-fold diluted supernatants were used to measure IL-2, IL-5, IL-7, IL-10, IL-12(p70) and IL-13 (low level cytokines). These cytokines has previously been identified as those of most relevance after stimulation of PBMC from tularemia vaccinees with specific antigen derived from *F. tularensis* (Eneslätt et al., 2012). Estimated cytokine concentrations outside the range of the standard curve were censored to the nearest standard value. Samples were analyzed in duplicate.

### Flow cytometry analysis of surface markers and intracellular cytokine staining

After 72 h of co-culture, non-adherent cells were transferred to a new plate and 5 μg/mL of Brefeldin A was added. Four-hours later, plates were centrifuged for 3 min at 500 × *g* and supernatants were removed. Cells were prepared for labeling with cell surface marker monoclonal antibodies (mAb) or conjugated intracellular cytokine mAb as recommended by BD Biosciences. The following mAb conjugates were used: CD3-APCCy7 (clone SK7, BD Biosciences), CD4-PE Texas red (clone S3.5, Caltag/Invitrogen), CD8-PerCPCy5.5 (clone SK1, BD Biosciences), IFNγ-FITC (clone 25723.11, BD Biosciences), MIP-1β-PE (clone D21-1351, BD Biosciences), CD107a-APC (clone H4A3, BD Biosciences), TNF-Brilliant violet 421 (clone MAb11, BioLegend), IL17A-Alexa F700 (clone N49-653, BD Biosciences). Aqua Viability Dye (Molecular Probes/Invitrogen) was added to distinguish live and dead cells. Cells were acquired using an LSRII flow cytometer (BD Biosciences) with FACSDiva software (BD Biosciences). Results were analyzed using FlowJo software (Tree Star).

### Data analysis and statistical methods

Wilcoxon's rank-sum test or Student's *t*-test were used to identify significant differences (*P* < 0.05) between data sets. Spearman's rank correlation with a 0.05 significance level was used to test whether two variables were correlated. A significant correlation with a coefficient above 0.4 was considered a strong association, and above 0.7 a very strong association.

For vaccinated individuals, the cytokine levels (cl) were linearly dependent on the CFU (data not shown). Therefore, all cytokine concentrations were normalized as follows. For each cytokine, data (CFU and cl observations) from the vaccinees were selected and linear regression was used to model the relationship between CFU and expected cytokine levels (ecl), which resulted in a model *ecl* = α + β*CFU*. This model was then used to calculate the *normalized cytokine levels* (ncl), where *ncl* = *cl* + *ecl*.

## Results

### Optimization of conditions for intracellular bacterial assay

Adherent cells were infected with LVS or SCHU S4 at various MOIs of 1:1, 10:1, and 100:1 (bacteria:adherent cell). Maximal control of bacterial replication occurred when the MOI of 10:1 was used, although significant control also was observed for the other two MOIs. Since only one MOI could be used for practical reasons, the 10:1 ratio was chosen for all presented experiments. The ratio of effector vs. target cells was also investigated using a range from 1:1 to 25:1. Consistently, the ratio 20:1 was found to confer maximal control of bacterial replication.

### Composition of effector cells used in assay of intracellular growth inhibition

**Figure 1:**
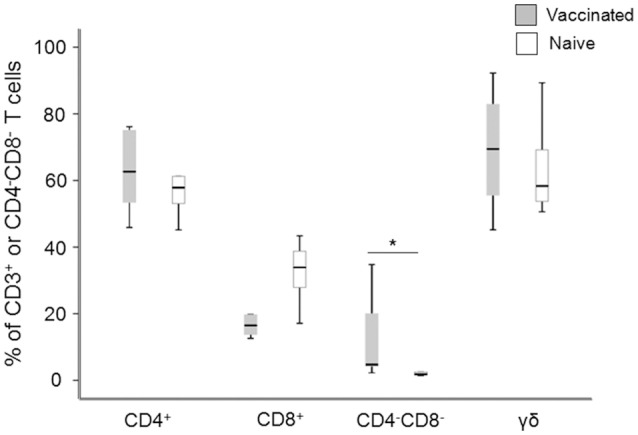
Flow cytometry analysis of the composition of the non-adherent cells after 6 d of antigen stimulation. The values for the CD4^+^, CD8^+^, and CD4^−^CD8^−^ cells are expressed as percentages of the total number of CD3^+^ T cells and the values for γ/δ T cells are expressed as percentages of the total number of CD4^−^CD8^−^ T cells. Levels of CD4^−^CD8^−^ T cells were significantly higher in vaccinees compared to naïve individuals (^*^P < 0.05). The line through each box shows the median, with quartile one and three as the lower and upper limits of each box. The end of the vertical lines indicates maximum and minimum values, respectively.

PBMC from naïve individuals and vaccinees were stimulated with specific *F. tularensis* antigen and after 6 days of culture, cells were characterized with regard to cell surface markers. 90–95% of the non-adherent cells were CD3^+^ cells and a majority of these, around 60%, were CD4^+^, whereas CD8^+^ T cells constituted between 20 and 30% with no differences between vaccinees and naïve individuals (Figure 1). Apart from the classical single-positive T cells, we identified significantly higher percentages of CD3^+^CD4^−^CD8^−^ cells in recall-stimulated cultures from vaccinees compared to naïve individuals [4.7% vs. 1.8% of CD3^+^ T cells (Figure 1)], whereas very few cells were CD3^+^CD4^+^CD8^+^ cells (< 1% of CD3^+^ T cells). Among the CD3^+^CD4^−^CD8^−^ cells, >50% were γδ^+^ T cells, but with no differences among the groups (Figure 1).

### Proliferative responses to *F. tularensis* antigens

**Figure 2:**
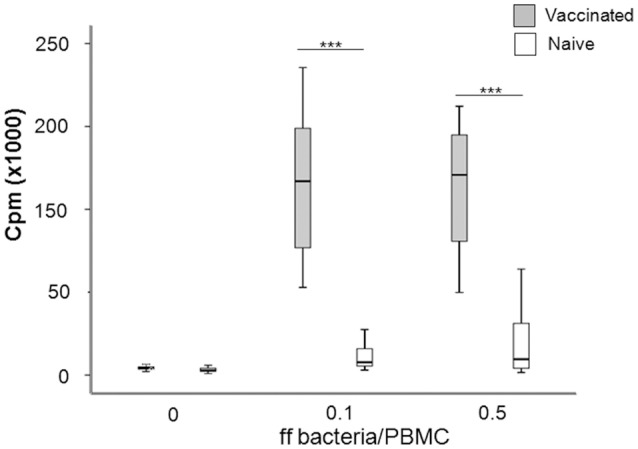
Box plot showing the proliferative responses of PBMC from naïve and vaccinated donors to recall antigen stimulation. Proliferation was measured by incorporation of [^3^H]-thymidine upon stimulation with indicated concentrations of ff bacteria/PBMC for 5 days (^***^P < 0.0001). The line through each box shows the median, with quartile one and three as the lower and upper limits of each box. The end of the vertical lines indicates maximum and minimum values, respectively.

In order to characterize the immune response of the individuals, PBMC were isolated from vaccines or naïve individuals and their proliferative responses to recall stimulation with formalin-fixed *F. tularensis* antigen (ffFt) were measured. The proliferative responses of PBMC from vaccinees were significantly higher (*P* < 0.001) than of PBMC from naïve individuals; this difference was seen irrespective of antigen concentration (Figure 2). Although PBMC from naïve individuals showed an increase in proliferation with increasing antigen concentration, this difference was not significant (Figure 2). The responses to a mitogen, ConA, were very similar between the two groups; 26,100 cpm ± 10,600 for naïve individuals vs. 21,300 cpm ± 5,500 for vaccinees (Spearman's correlation coefficient *P* > 0.60).

In summary, the results showed that the immune individuals showed significantly higher *F. tularensis*-specific proliferative responses, as expected from their immune status.

### Control of intracellular replication of *F. tularensis* and cytokine secretion by PBMC

**Figure 3:**
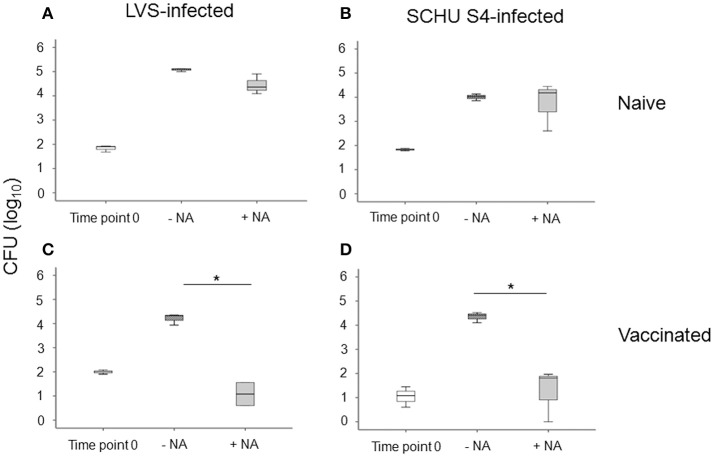
Growth inhibition of LVS-infected PBMC cultures **(A,C)**, or SCHU S4-infected cultures **(B,D)**. PBMC were isolated from a naïve **(A,B)** or from a vaccinated individual **(C,D)**. After 72 h of co-culture with adherent and non-adherent cells (NA), the number of intracellular bacteria was determined. Results are from triplicate wells of a representative donor for each group (^*^P < 0.05). Time point 0 indicates the bacterial numbers after uptake and washing. –NA indicates the bacterial numbers in cultures without non-adherent cells after 72 h. +NA indicates the bacterial numbers in cultures with non-adherent cells after 72 h. The line through each box shows the median, with quartile one and three as the lower and upper limits of each box. The end of the vertical lines indicates maximum and minimum values, respectively.

**Figure 4:**
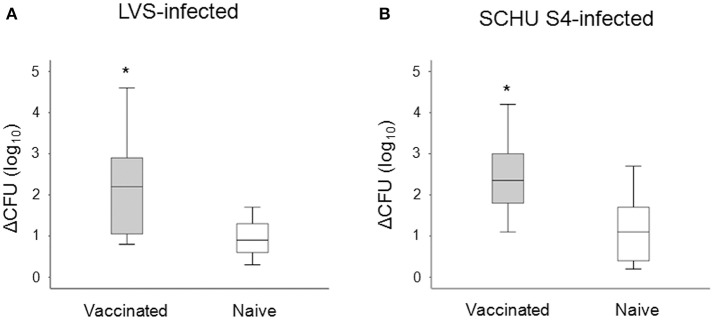
Growth inhibition of LVS-infected **(A)**, or SCHU S4-infected cultures **(B)**. After 72 h of co-culture with adherent and non-adherent cells, the number of intracellular bacteria was determined and the delta CFU (log_10_) was calculated as CFU (log_10_) of cultures without non-adherent cells subtracted with the CFU (log_10_) of cultures with non-adherent cells. The delta CFU (log_10_) was significantly higher in vaccinated individuals compared to naïve individuals for both LVS-infected and SCHU S4-infected cultures (^*^P < 0.05). Results represent data from 11 individuals of each group and each group's median is illustrated by the line through each box, with quartile one and three as the lower and upper limits of each box. The end of the vertical lines indicates maximum and minimum values, respectively.

We investigated the potential of PBMC to control the intracellular replication of SCHU S4 or LVS in cell cultures. To this end, non-adherent PBMC were stimulated with specific *F. tularensis* antigen for 6 days, counted and checked for viability, typically 80–95% viable cells, and thereafter added to cultures with LVS- or SCHU S4-infected, autologous, adherent PBMC using the aforementioned optimal MOI and target/effector ratios. The bacterial uptake was very similar regardless of whether the adherent cells originated from vaccinees or naïve individuals (*P* > 0.84). After 72 h of co-culture with target and effector cells, the number of intracellular bacteria was determined and the differences (log_10_ CFU) in bacterial numbers in the cultures without non-adherent cells vs. the cultures with non-adherent cells were calculated. A representative experiment is shown in Figure 3 illustrating that addition of non-adherent cells from a naïve individual did not confer any significant control of LVS (Figure 3A) or SCHU S4 bacteria (Figure 3B), whereas the addition of non-adherent cells from a vaccinee resulted in significant differences (*P* < 0.001), approximately 3 log_10_ lower bacterial numbers, than in the absence of non-adherent cells (Figures 3C,D). When groups of individuals were analyzed, it was observed that there was highly significant control of bacterial replication in the presence vs. the absence of non-adherent PBMC from vaccinees (*n* = 11); *P* < 0.005 for cultures with LVS-infected or SCHU S4-infected cells, whereas addition of non-adherent PBMC from naïve individuals (*n* = 11) did not result in significant control (*P* > 0.05) of bacterial numbers in any of the cultures (data not shown). Overall, the control exerted by PBMC from vaccinees was significantly greater vs. than that executed by PBMC from naïve individuals, *P* = 0.025 for LVS-infected cultures and *P* = 0.011 for SCHU S4-infected cultures (Figure 4). Thus, control of intracellular bacterial replication correlated to the vaccination status of the donors.

**Figure 5:**
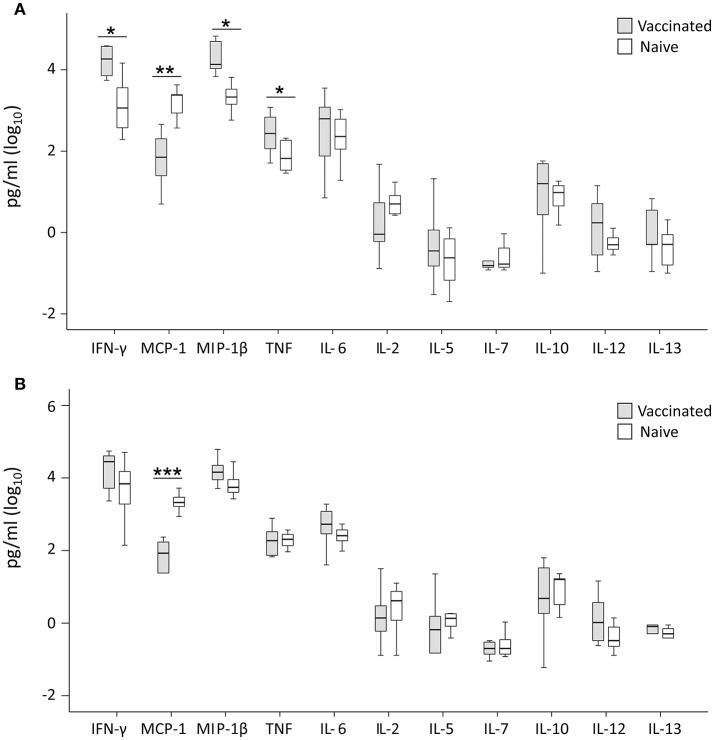
Levels of secreted cytokines in supernatant of T cells co-cultured for 72 h with LVS-infected **(A)** or SCHU S4-infected **(B)** adherent cells from vaccinated (gray) or naïve (white) individuals. Levels of IFN-γ, MIP-1β and TNF were significantly higher in the LVS-infected cultures with cells from vaccinated individuals (^*^P < 0.05), whereas no significant differences were observed for the SCHU S4-infected cultures. The levels of MCP-1 were significantly higher in cultures with cells from naïve individuals regardless of strain used (^**^P < 0.01; ^***^P < 0.001). Results represent data from 11 individuals of each group and each group's median is illustrated by the line through each box, with quartile one and three as the lower and upper limits of each box. The end of the vertical lines indicates maximum and minimum values, respectively.

Cytokine levels were determined for 11 cytokines and levels compared between cultures with PBMC from vaccinees vs. naïve individuals. No significant differences were observed for IL-2, IL-5, IL-7, IL-10, IL-12, or IL-13 between the two groups, regardless of whether the cultures had been infected with LVS or SCHU S4. IFN-γ, MIP-1β, TNF, were consistently higher in LVS-infected cultures with PBMC from vaccinees vs. cultures with cells from naïve individuals (*P* < 0.05 for IFN-γ and TNF and *P* < 0.01 for MIP-1β; Figure 5A). Also, in SCHU S4-infected cultures, levels of IFN-γ and MIP-1β were higher in vaccinees vs. naïve individuals, however, the differences were non-significant (*P* > 0.05; Figure 5B). Regardless of infecting strain, the levels of MCP-1 were significantly higher with PBMC from naïve individuals compared to PBMC from vaccinees (*P* < 0.005 with LVS, *P* < 0.0005 with SCHU-S4).

**Figure 6:**
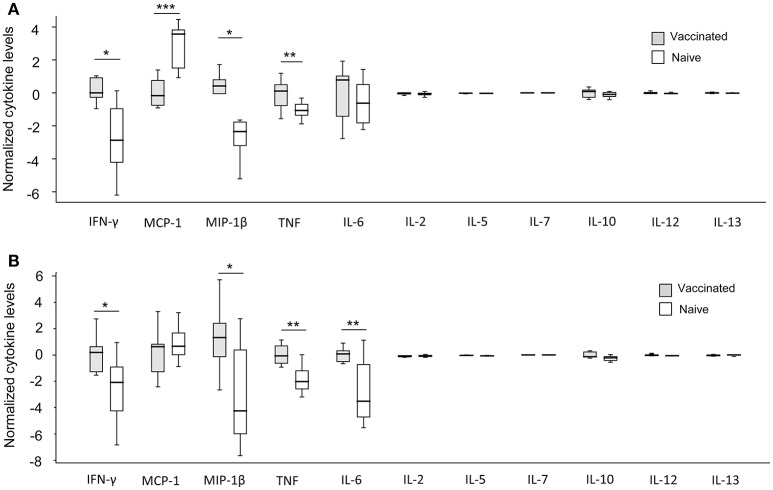
Normalized cytokine levels in LVS- **(A)** and SCHU S4- **(B)** infected co-cultures with PBMC from vaccinated (gray) or naïve (white) individuals. Levels of IFN-γ, MIP-1β, and TNF were significantly higher in cultures regardless of infecting strain, while IL-6 levels were significantly higher only in SCHU S4-infected cultures (^*^P < 0.05; ^**^P < 0.01; ^***^P < 0.001). The line through each box shows the median, with quartile one and three as the lower and upper limits of each box. The end of the vertical lines indicates maximum and minimum values, respectively.

When absolute cytokine levels were normalized for CFUs, the normalized levels of IFN-γ, MIP-1β, and TNF were significantly higher in both LVS- and SCHU S4-infected cultures with PBMC from vaccinees than with PBMC from naïve individuals (*P* < 0.05 for IFN-γ and MIP-1β and *P* < 0.01 for TNF; Figure 6). IL-6 levels were significantly higher (*P* < 0.01) in SCHU S4-infected cultures with PBMC from vaccinees vs. naïve individuals (Figure 6B), whereas the levels of IL-6 did not differ in LVS-infected cultures (Figure 6A). Thus, levels of IFN-γ and MIP-1β served as correlates of immunity, since they discriminated between vaccinees and naïve individuals.

Thus, the normalized cytokine levels more frequently demonstrated significant differences and also lower *P*-values between the groups (Figure 6), than did the actual cytokine levels in the supernatants, in particular with regard to the SCHU S4-infected cultures (Figure 5). This indicates that the bacterial numbers *per se* affect the cytokine levels.

### Intracellular cytokine levels of T cells in the co-culture assays

After 72 h of incubation, the non-adherent cells from LVS infected co-cultures were analyzed for intracellular cytokines by flow cytometry. CD3^+^CD4^+^ T cells from vaccinees showed significantly higher level of IFN-γ, MIP-1β, TNF, and CD107a, but not IL-17, compared to the same cells from naïve individuals (*P* < 0.05; Table 1), but there were no significant differences between the two groups with regard to the CD3^+^CD8^+^ T cells (data not shown).

**Table 1:** Intracellular cytokine level expression by CD4 T cells in co-culture assay.

	**CD4 T cells**				
	**IFN-γ^a^**	**IL-17**	**MIP-1β**	**TNF**	**CD107a**
Vaccinated	0.8 ± 0.2^b^^*^	0.3 ± 0.1	2.5 ± 0.6^*^	1.3 ± 0.3^*^	1.4 ± 0.3^*^
Naïve	0.3 ± 0.1	0.2 ± 0.0	0.2 ± 0.0	0.4 ± 0.0	0.4 ± 0.1

a *Intracellular cytokine staining and flow cytometry analysis of cells were performed after 72 h of incubation in the co-culture assay infected with the LVS strain*.

b *Data represent mean percentages ± SEM (n = 8)*.

* *P < 0.05 vs. the group of naïve individuals*.

## Discussion

Immunity against intracellular pathogens is often critically dependent on CMI. This hampers the identification of correlates of immunity and protection to such pathogens, since there are no validated methods for this identification. The lack of methods also hamper vaccine development, since they are required for the fulfillment of the Animal Rule. Moreover, since tularemia is rather infrequently occurring in most parts of the world, human clinical trials will likely not confer sufficient statistical significance for validation of efficacy and the Animal Rule is likely the only option for licensing of future tularemia vaccines. This option is most likely applicable to biodefense agents and sporadically occurring diseases, both of which are relevant to tularemia. However, methodological developments will be required to overcome the obstacles before the requirements of the Animal Rule can be fulfilled. These require that efficacy in animal models of relevance will be compared to a model that establishes human correlates of immunity and protection (Snoy, 2010). The latter will require bactericidal effects as surrogate measures of vaccine efficacy and we therefore sought to establish a model that would fulfill this criterion and the present study was designed accordingly.

A lack of correlates of immunity is not unique to tularemia, for example no validated method for identification of correlates exists for the extremely common disease tuberculosis (Nguipdop Djomo et al., 2013). Although patterns of polyfunctional cytokine-producing T cells have been proposed to correlate with tuberculosis vaccine efficacy (Derrick et al., 2011), these patterns are similar regardless of age, despite that vaccination with BCG confers better protection in children than in adults (Colditz et al., 1994; Kagina et al., 2010); thereby questioning their relevance. Such polyfunctional T cells have been identified also for tularemia, e.g., during human recall responses after LVS vaccination, secretion of IL-12, IFN-γ, MCP-1, MIP-1β, IL-17, and IL-22 have been identified, which served as immuno-specific signatures and discriminated between immune and naïve individuals (Paranavitana et al., 2010; Eneslätt et al., 2012). Much work has been performed based on mouse models of tularemia in order to identify correlates of immunity (Cowley et al., 2007; Elkins et al., 2007; Cowley and Elkins, 2011; Ryden et al., 2012) and, again, secreted Th1-related cytokines, such as IFN-γ, TNF, and MCP-1, were observed and found to correlate to the protective efficacies obtained after immunization with attenuated mutants of *F. tularensis* subspecies *tularensis* (Ryden et al., 2012). In another study, the cytokine gene expression of leukocytes derived from lung, liver, and spleen was examined following immunization with variants of LVS that show variable protective efficacies. The overall pattern was complex when statistical models were developed to predict vaccine efficacy, however, several Th1 cytokine-associated factors were in combinations strongly predictive of protective efficacy, e.g., TNF, IFN-γ, T-bet, IL-27, and IL-12Rβ2 (De Pascalis et al., 2014). In a recent study based on co-culture model using spleen cells from immunized mice, levels of nitric oxide, IFN-γ, IL-17, and GM-CSF strongly correlated with control of intramacrophage infection with SCHU S4 (Golovliov et al., 2017). Altogether, although some markers of immunity have been identified, their relevance for protection is generally poorly understood and there is a need of data that more directly demonstrate correlation to protection.

*Francisella tularensis* clearly is able to infect many cell types *in vivo*, however, the cumulative evidence indicates that the macrophage is a key target for the infection (Elkins et al., 2007). The present human co-culture system was established with the aim to model *in vivo* interactions between infecting bacteria, their target host monocytic cells, and effector lymphocytes, with the assumption that bacterial replication would be restricted due to the interactions as has been shown in a murine co-culture system (Elkins et al., 2011). The non-adherent PBMC from immune individuals were stimulated with *F. tularensis* antigens to selectively expand antigen-specific, memory-immune T cells, whereas PBMC from naïve individuals were stimulated to control for nonspecific innate immunity and bystander effects. Cells from immune individuals generally showed efficient control of bacterial replication and, interestingly, this effect was essentially the same, whether or not the LVS strain or the SCHU S4 strain was used. In contrast, cells from naïve individuals showed minimal or no control of the *F. tularensis* infection. In view of this background, we hypothesized that the memory T-cell-mediated inhibition of intracellular *F. tularensis* observed in the co-culture model would correlate to the occurrence of Th1 cytokines and, thus, the latter would serve as correlates for protection to tularemia. Indeed, several pieces of evidence indicate that this, in fact, was the case. Thus, the present findings that normalized levels of IFN-γ, TNF, and MIP-1β and absolute levels of IFN-γ and MIP-1β in LVS-infected cultures correlated with the degree of protection observed in the cultures are in agreement with the hypothesis and are therefore of potential relevance as protective correlates. In addition, we previously demonstrated that IFN-γ and MIP-1β were expressed by CD4^+^CD45RO^+^ and CD8^+^CD45RO^+^ human T cells (Eneslätt et al., 2012), the same cytokines expressed by CD4 T cells were also identified in the present study as discriminating between naïve and immune individuals. It should be noted that the variation in cytokine levels within the groups of vaccinated or naïve individuals was substantial and in some instances rendered marked differences between the groups non-significant. Presumably, this is a reflection of the normal variation of an out-bred population. Interestingly, the variation within the groups was less marked with regard to the control of infection.

To identify human immune correlates of tularemia, several obstacles must be avoided, one of which is the almost complete absence of detectable levels of circulating cytokines in tularemia patients. In a previous study, sera were obtained from patients at five time points up to 4 weeks after onset of tularemia, but of eight cytokines studied, only IFN-γ was detected and only transiently on day two (Andersson et al., 2006). Moreover, if immunospecific signatures are identified by *in vitro* studies, it cannot be determined if they simply are correlates, or also provide mechanistic information. Thus, it will be important to complement such findings with assays, such as the co-culture model, that will provide mechanistic insights, thus fulfilling the requirements of the Animal Rule. An additional strength of the co-culture model is that similar murine models exist and they have been used to identify transcriptional signatures that discriminate *Francisella* vaccines of different efficacies and thus could serve as potential correlates of protection (De Pascalis et al., 2012, 2014; Mahawar et al., 2013; Griffin et al., 2015; Golovliov et al., 2017).

There is a multitude of evidence for the critical role of IFN-γ for immunity to *F. tularensis* (Anthony et al., 1989; Surcel et al., 1991; Fortier et al., 1992; Conlan et al., 1994; Elkins et al., 1996; Sjöstedt et al., 1996; Cowley et al., 2010) and this was further corroborated by our present findings, since the *in vitro* growth control detected in the co-culture model correlated to secretion of IFN-γ. Effector mechanisms have been much studied for the ability of macrophages to control *F. tularensis* infection in the murine model (Cowley and Elkins, 2011). IFN-γ activation of macrophages effectively restricts the intracellular multiplication of *F. tularensis* and this is conferred by mechanisms involving both reactive nitrogen species (RNS), such as NO, and reactive oxygen species (ROS), whereas their roles are less evident with regard to virulent strains (Lindgren et al., 2004a,b, 2007; Santic et al., 2005; Bönquist et al., 2008; Edwards et al., 2010; Mahawar et al., 2013; Griffin et al., 2015). We recently demonstrated, however, that NO strongly correlated with control of both LVS and SCHU S4 infection in a mouse co-culture model (Golovliov et al., 2017). The IFN-γ-mediated control of the closely related bacterium *F. novicida* is strictly dependent on the IFN-γ-inducible guanylate-binding proteins GBP2 and GBP5 (Man et al., 2015; Meunier et al., 2015) and this is also true for the LVS strain, but not for the SCHU S4 strain (Wallet et al., 2017). Thus, evidence from animal models indicate that the control of highly virulent strains is distinct from that of attenuated *F. tularensis* strains and demonstrate that the use of such strains in the co-culture models is necessary to identify relevant correlates of immunity and protection.

The effective control effectuated in the present co-culture model based on the highly virulent SCHU S4 strain is important, since it implicates that it should be possible to achieve human vaccine-mediated immune responses that effectively control natural infections evoked by other strains of the highly virulent subspecies *tularensis*. In this regard, it is noteworthy that a likely limitation of the widely used mouse model for evaluation of *F. tularensis* vaccines is the exquisite susceptibility of mice, since regardless of route of infection; the lethal dose of virulent *F. tularensis* strains is one bacterium (Lyons and Wu, 2007).

A limitation of the present study was the exclusive use of PBMCs, since it is unknown how well these types of cells mimic the phenotypes of tissue-localized immune cells. In the mouse model, there is ample evidence that vaccination induces qualitatively distinct immune responses in different organs, for example, in a mouse model of tuberculosis, vaccination with BCG and then an intradermal boost with a recombinant antigen led to strong splenic Th1 T cell responses, whereas an intranasal boost resulted in efficacious control of an aerosol challenge, but weak splenic responses (Forbes et al., 2008). Likewise, a study on *Francisella* demonstrated that a variant of LVS conferred better protection against an intraperitoneal challenge after intranasal than after intradermal vaccination (De Pascalis et al., 2014). The results suggest that there are organ-specific qualitative differences between immune cells and, therefore, the route of immunization may be important to achieve optimal protection and also that the source of cells used in the *in vitro* systems matters. However, in the case of humans, there are few alternative cell sources and peripheral blood cells are the only readily available cell type. Accordingly, there a several examples of similar *in vitro* co-culture systems based on the use of peripheral blood cells developed for the use of evaluating vaccine efficacy and defining protective correlates against tuberculosis (Hoft et al., 2002; Parra et al., 2009; Berry et al., 2010; Bloom et al., 2012).

We and others have implemented logistic modeling to investigate the multivariate relations between the different types of correlates that can be identified *in vivo* and using co-culture systems, such as secreted cytokines, cytokine gene expression, bacterial numbers, lymphocyte stimulation indices etc. (Eneslätt et al., 2011, 2012; Ryden et al., 2012; De Pascalis et al., 2014). Previously, we implemented it to build models based on human *ex vivo* data that with the smallest number of features and the highest accuracy predicted the immune status of a donor (Eneslätt et al., 2011). Based on mouse data, a publication combined results from the co-culture method and gene expression *in vivo* and combining the two are likely desirable since they should have complementary properties (De Pascalis et al., 2014). By performing similar types of analyses based on data from multiple sources, we envision that it will be feasible to make inter-tissue and interspecies comparisons. This type of modeling may help to identify individual candidate correlates so that their relevance can be investigated in each of the systems (Kurtz et al., 2013; Melillo et al., 2013) with the ultimate aim that they will represent a rational strategy to evaluate vaccines by monitoring the correlates post-immunization as a first step to achieve the requirements of the Animal Rule.

Today, all licensed vaccines are based on correlates of protection derived from measures of humoral immunity and since many of them are based on relatively technically straightforward assays utilizing serum, they are convenient to use (Nguipdop Djomo et al., 2013). The rational development of vaccines that predominantly trigger CMI will require the implementation of similarly simple methods to gauge the correlates; however, all of this work is still in its infancy and the present work on human correlates for tularemia is a first step to identify such correlates.

## Author contributions

KE, AS: Conceived and designed the experiments; KE, IG: Performed the experiments; KE, IG, PR, AS: Analyzed the data; KE, PR, AS: Wrote the paper.

### Conflict of interest statement

The authors declare that the research was conducted in the absence of any commercial or financial relationships that could be construed as a potential conflict of interest.
